# Risk and protective factors for falls on stairs in young children: multicentre case–control study

**DOI:** 10.1136/archdischild-2015-308486

**Published:** 2015-12-10

**Authors:** D Kendrick, K Zou, J Ablewhite, M Watson, C Coupland, B Kay, A Hawkins, R Reading

**Affiliations:** 1 Division of Primary Care, School of Medicine, Nottingham, UK; 2 School of Health Sciences, University of Nottingham, Queen's Medical Centre, Nottingham, UK; 3 Emergency Department, Bristol Children's Hospital, Bristol, UK; 4 Great North Children's Hospital, Newcastle upon Tyne Hospitals NHS Foundation Trust, Research Unit Level 2, Newcastle upon Tyne, UK; 5 Jenny Lind Paediatric Department, Norfolk and Norwich University Hospital, Norfolk Community Health and Care NHS Trust, Norwich, UK

**Keywords:** falls, stairs, risk factors, case-control

## Abstract

**Aim:**

To investigate risk and protective factors for stair falls in children aged <5 years.

**Methods:**

Multicentre case–control study at hospitals, minor injury units and general practices in and around four UK study centres. Cases were children with medically attended stair fall injuries. Controls were matched on age, sex, calendar time and study centre. A total of 610 cases and 2658 controls participated.

**Results:**

Cases’ most common injuries were bangs on the head (66%), cuts/grazes not requiring stitches (14%) and fractures (12%). Parents of cases were significantly more likely not to have stair gates (adjusted OR (AOR) 2.50, 95% CI 1.90 to 3.29; population attributable fraction (PAF) 21%) or to leave stair gates open (AOR 3.09, 95% CI 2.39 to 4.00; PAF 24%) both compared with having closed stair gates. They were more likely not to have carpeted stairs (AOR 1.52, 95% CI 1.09 to 2.10; PAF 5%) and not to have a landing part-way up their stairs (AOR 1.34, 95% CI 1.08 to 1.65; PAF 18%). They were more likely to consider their stairs unsafe to use (AOR 1.46, 95% CI 1.07 to 1.99; PAF 5%) or to be in need of repair (AOR 1.71, 95% CI 1.16 to 2.50; PAF 5%).

**Conclusion:**

Structural factors including having landings part-way up the stairs and keeping stairs in good repair were associated with reduced stair fall injury risk. Family factors including having stair gates, not leaving gates open and having stair carpets were associated with reduced injury risk. If these associations are causal, addressing these factors in housing policy and routine child health promotion could reduce stair fall injuries.

**What is already known on this topic**

- Falls are the leading cause of medically attended injury in preschool children, and falls from stairs comprise 12% of hospital admissions and 18% of emergency department attendances for falls.
- Home safety education and equipment provision can increase use of stair safety gates, but there is little evidence that this reduces injuries.

**What this study adds**

- Family factors including use of stair gates, not leaving them open and having a stair carpet were associated with reduced risk of a stair fall injury.
- Structural factors including having a landing part-way up the stairs and keeping stairs in good repair were associated with reduced injury risk.
- If these associations are causal, addressing these factors in routine child health promotion and housing policy could reduce stair fall injuries.

## Introduction

Falls are the leading cause of medically attended injury in children aged <5 years in most high-income countries.^1^ In England and Wales, in 2002, the latest year for which detailed emergency department (ED) data is available, falls among the children aged <5 years resulted in more than 190 000 ED attendances,^2^ and in 2012/2013 they accounted for almost 20 000 hospital admissions in England.^3^ Falls from stairs or steps comprised 18% of ED attendances for falls^2^ and 12% of admissions for falls.^3^ While some falls from stairs among children aged <5 years are associated with objects such as baby walkers, toys or pushchairs, most (88%) are not and only a small proportion arise from children being dropped while being carried on stairs.^4^ A recent systematic overview found interventions providing home safety education, and/or home safety equipment were effective in promoting the use of safety gates on stairs and some evidence that they reduce the number of families using baby walkers. The overview found little evidence that these interventions reduced injury rates.^5^ Our study aimed to quantify risk and protective factors for stair falls among children aged <5 years.

## Methods

The published protocol reports full details of the methods.^6^ This study was one of five concurrent case–control studies, each recruiting children with one type of injury (falls from furniture, falls on one level, stair falls, poisoning, scalds).

### Study design and setting

The study was conducted in NHS hospitals in Nottingham, Bristol, Newcastle upon Tyne, Norwich, Gateshead, Derby, Lincoln and Great Yarmouth, England. Case recruitment commenced on 14 June 2010 and ended on 30 September 2012. Control recruitment commenced at the same time as case recruitment and ended within 4 months of case recruitment.

### Participants

Cases were children aged 0–4 years attending EDs, minor injury units or admitted to hospital following a fall on stairs in the child's home. Children with intentional or fatal injury or living in residential care were excluded. Parents/carers of potentially eligible children were invited to participate during their medical attendance or by telephone or post within 72 h of attendance.

Controls were children aged 0–4 years without a medically attended stair fall, recruited from the case's general practice (or neighbouring practice). We aimed to recruit an average of four controls for each case matched on age (up to 4 months younger or 4 months older than the case), gender and calendar time (recruited up to 4 months of the date of the case injury). Study invitations were sent to 10 potentially eligible controls for each case by mail from the practice register. Where more than 10 control participants met inclusion criteria, those with dates of birth closest to that of their matched case were chosen. To increase power and make efficient use of recruited participants, control participants from cases with more than four controls, controls no longer matched to cases (eg, case had subsequently been excluded) and control participants from the other four ongoing case–control studies were matched (on study centre, age, gender and calendar time) to cases which had fewer than four controls.

Participating parents/carers completed age-specific (0–12, 13–36 and ≥37 months) questionnaires. One reminder was used for non-responders. Those completing questionnaires were given a £5 gift voucher. Other methods, shown in a systematic review to increase response rates, were used, including personalised invitations, first class mailings, reminders and inclusion of university logos on study documentation.^7^ Questionnaires collected data on exposures, socio-demographical and confounding variables, injuries and treatment received.

### Sample size

Sample size was based on 80% power, 5% significance level, four controls per case and a correlation between exposures in cases and matched controls of 0.1. To detect an OR of 1.43, 496 cases and 1984 control participants were required based on prevalence of exposures from previous studies (baby walker use (36%), no safety gates on stairs (55%), not using playpens (58%) and not using stationary activity centres (76%)).^8^ ^9^

### Exposures

The exposures of interest were safety behaviours, safety equipment and home hazards relating to stairs. These are described in table 1, with reporting periods, response options and response categorisations.

**Table 1:** Exposures and reporting periods, response options and categorisation of responses

Exposures reported 24 h prior to injury (cases) or 24 h prior to completing questionnaire (controls)	Response option	
Safety gates		
a. Used anywhere in the home	Yes/no	
b. Used on stairs	Yes/no	Grouped into: closed stair gate/gate left open/no gate
c. Left stair gate open	Yes/no	
Stair features		
a. Landing part-way up	Yes/no	
b. Spiral stairs or winding stair cases	Yes/no	
c. Handrails on stairs	On all stairs/on some stairs/no	
d. Banister/railing on stairs	On all stairs/on some stairs/no Grouped into: on all stairs vs other responses	
e. Banister/railing: width of biggest gap	Inches Grouped into: tertiles	
f. Stair covering	Carpet/wood/metal/concrete/lino/vinyl/don't know/other Grouped into: carpet vs other responses	
g. Stairs are too steep	Agree/neither agree or disagree/disagree	
h. Stairs are too narrow	Agree/neither agree or disagree/disagree	
i. Stairs are poorly lit	Agree/neither agree or disagree/disagree	
j. Steps are in need of repair	Agree/neither agree or disagree/disagree	
k. Banister/handrail is in need of repair	Agree/neither agree or disagree/disagree	
l. Stair covering is in need of repair	Agree/neither agree or disagree/disagree	
m. Stairs are safe to use	Agree/neither agree or disagree/disagree Grouped into: agree vs other responses A composite stair safety variable (for use as a confounder in analyses) included items (g) to (m) grouped as: No safe responses: agree to all of items (g) to (l) and disagree to (m) All safe responses: disagree to all items (g) to (l) and agree to item (m) Some safe responses: all other combinations of responses	
Use of baby walkers (ages 0–36 months only)	Yes/no	
Use of playpens or travel cots while child awake (ages 0–36 months only)	Yes/no	
Use of stationary activity centres (ages 0–36 months only)	Yes/no	

**Exposures reported for 1 week prior to injury (cases) or 1 week prior to completing questionnaire (controls)**	**Response option**	

Tripping hazards on stairs	Every day/most days/some days/never/does not apply Grouped into: at least some days vs never	

**Exposures ever reported prior to injury (cases) or prior to completing questionnaire (controls)**	**Response option**	

Taught child safety rules or instructions about		
a. How to behave when going down the stairs	Yes/no	
b. Carrying big/lots of things while going down the stairs	Yes/no	
c. Leaving things on stairs	Yes/no	

### Confounders

All analyses accounted for matching by age and sex and adjusted for distance from home residence to hospital (categorised into quintiles)^10^ and Index of Multiple Deprivation (IMD, linear term).^11^ Distance from residence to hospital and IMD were included because some control participants came from very different neighbourhoods than case participants and the extra matched controls were not matched on practice. Distance from residence to hospital was grouped into quintiles (≤2.0, 2.1–3.2, 3.3–4.7, 4.8–8.8, >8.8 km). Directed acyclic graphs were constructed for each exposure to identify the minimal sufficient adjustment set of confounders that analyses needed to adjust for.^12^ Potential confounders entered into directed acyclic graphs were first child (yes/no); overcrowding (yes/no); ethnic group (white/other); single adult household (yes/no); the Hospital Anxiety and Depression Scale (HADS, linear term)^13^; Parenting Daily Hassles Scale (PDH, linear term)^14^ ^15^; child behaviour questionnaire score (linear term)^16–18^; hours of out-of-home child care per week (linear term) and child's ability to open safety gate (likely/not likely). Some exposures were also considered as potential confounders for other exposures including use of playpen, teaching safety rules on stairs, stair gates and the composite stair safety variable described in table 1. Analyses for each exposure were adjusted for those confounders identified in the directed acyclic graphs as being in the minimal sufficient adjustment set (listed in table 3).

### Statistical analysis

Associations between exposures and stair falls were estimated using ORs and 95% CIs, using conditional logistic regression adjusted for confounders as described above. The linearity of relationships between continuous confounders and case/control status was tested by adding higher-order terms to regression models, with categorisation where there was significant non-linearity. Interaction terms were added to regression models to explore differential effects by child age, gender, ethnic group, single parenthood, non-owner-occupied housing and unemployment. An interaction between use of baby walkers and use of safety gates on stairs was also examined. Significance of interactions was assessed with likelihood ratio tests (p<0.01) and stratified OR presented where significant interactions were found. The population attributable fraction (PAF) percentage was calculated for exposures with statistically significantly raised adjusted ORs (AORs) using a published formula.^19^

For the HADS, single missing item values for each subscale were imputed using the mean of the remaining six items. Subscale scores were not computed when more than one item was missing.^20^ The same approach was used for missing values of PDH, since we were unable to find specific guidance on this. The main analyses were complete case (CC) analyses including single imputed values for HADS and PDH. Cases and controls with responses of ‘not applicable’ were excluded from analyses where appropriate. Sensitivity analyses were performed using multiple imputation (MI), with case/control status, study centre, age and sex of child, IMD, distance from hospital, socio-demographical characteristics and all exposure and confounding variables included in the imputation model. This included imputation of HADS and PDH scores for cases and controls who had more than one item missing on these scales. Twenty imputed datasets were created and the results were combined using Rubin's rules.^21^

### Ethical approval

The study was approved by Nottinghamshire research ethics committee. Informed consent was assumed through return of completed study questionnaires.

## Results

**Figure 1:**
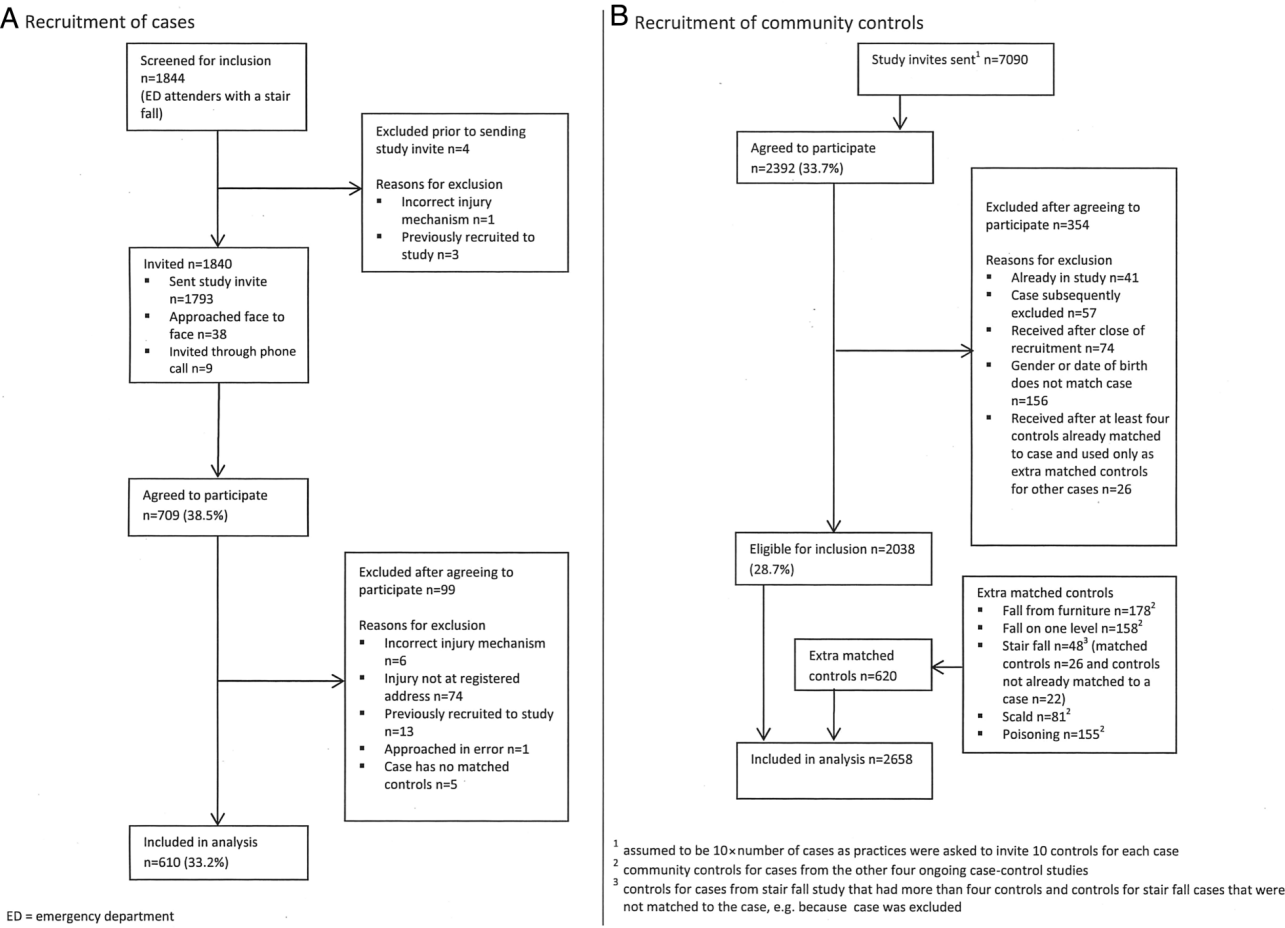
Flow of cases and controls through study (stair fall).

A total of 610 cases and 2658 controls participated as shown in figure 1. Thirty three percent of parents/carers of cases and 29% of controls agreed to participate. Child participants and non-participants were similar in terms of sex (50% vs 53% male), but a higher proportion of participants were aged 0–12 months than non-participants (19% vs 12%). The mean number of controls per case was 4.36. The median time from date of injury to date of questionnaire completion for cases was 11 days (IQR 7–21). Most cases sustained single injuries (85%), most commonly bangs on the head (66%), cuts/grazes not requiring stitches (14%) and broken bones (12%). Most cases (64%) were seen and examined but did not require treatment, 25% were treated in ED and 5% were admitted to hospital.

Table 2 shows that compared with controls, cases were less likely to live in a household with two or more adults in paid work (50% vs 59%). Cases also lived in more deprived areas (median IMD score 18.7 vs 15.2) and were more likely to live in single adult households (15% vs 11%), receiving state benefits (41% vs 32%), in non-owner-occupied housing (40% vs 32%), in households without a car (15% vs 10%) and with mothers who had their first child under the age of 20 years (19% vs 9%).

**Table 2:** Socio-demographical characteristics of cases and controls

Characteristics	Cases n=610 (%)	Controls n=2658 (%)
Study centre		
Nottingham	252 (41.3)	1055 (39.7)
Bristol	178 (29.2)	796 (29.9)
Norwich	97 (15.9)	457 (17.2)
Newcastle	83 (13.6)	350 (13.2)
Median child age (years) (IQR)*	2.0 (1.2–2.9)	2.0 (1.3–3.1)
Age in months*		
0-12	113 (18.5)	315 (11.9)
13–36	362 (59.3)	1694 (63.7)
37–62	135 (22.1)	649 (24.4)
Male	299 (49.0)	1320 (49.7)
Ethnic group white	547 (91.5) [12]	2371 (91.0) [52]
Number of children aged <5 years in family†	[8]	[44]
0	7 (1.2)	28 (1.1)
1	358 (59.5)	1566 (59.9)
2	212 (35.2)	911 (34.9)
≥3	25 (4.1)	109 (4.2)
First child	242 (43.3) [51]	1067 (44.5) [260]
Maternal age <20 at birth of first child‡	100 (18.5) [7]	219 (9.1) [15]
Single adult household	87 (14.6) [15]	272 (10.5) [76]
Median weekly hours out-of-home child care (IQR)	13.5 (1.0–22.5) [43]	15 (3.0–24.0) [165]
Adults in paid work	[16]	[56]
0	88 (14.8)	284 (10.9)
1	209 (35.2)	784 (30.1)
≥2	297 (50.0)	1534 (59.0)
Receives state benefits	241 (40.9) [21]	838 (32.4) [68]
Overcrowding (>1 person per room)	52 (9.1) [40]	187 (7.5) [152]
Non-owner-occupied housing	241 (40.4) [14]	836 (32.2) [65]
Household has no car	88 (14.7) [12]	254 (9.7) [50]
Median Index of Multiple Deprivation score (IQR)§	18.7 (10.1–32.7)	15.2 (9.0–27.1) [35]
Median kilometres from hospital (IQR)	3.4 (2.2–5.4)	3.9 (2.4–7.6) [34]
Mean Child Behaviour Questionnaire score (SD)§	4.7 (0.9) [43]	4.6 (0.9) [293]
Long-term health condition	63 (10.4) [6]	202 (7.6) [19]
Median Child Health Visual Analogue Scale (range 0–10) (IQR)§	9.9 (9.0–10.0) [9]	9.7 (8.4–10.0) [19]
Median Health-Related Quality of Life in children ≥2 years (PedsQL) (IQR)§¶	(n=303) [6] 91.7 (83.3–97.6)	(n=1342) [18] 89.3 (82.1–94.0)
Parental assessment of child's ability to open safety gate	[21]	[111]
Not likely	433 (73.5)	1937 (76.1)
Very or quite likely	156 (26.5)	610 (24.0)
Median Parenting Daily Hassles Tasks scale (IQR)§,**	14.0 (10.0–18.0) [61]	14.0 (11.0–18.0) [152]
Mean Hospital Anxiety and Depression Scale (SD)§,**	10.4 (6.2) [14]	10.7 (5.9) [36]

[ ] missing values.

*Age when questionnaire completed.

†Some families reported zero because children were aged <5 years at time of injury or at time of sending control questionnaire, but aged >5 years when questionnaire was completed.

‡Only applicable where mothers completed questionnaire.

§A higher Index of Multiple Deprivation score indicates greater deprivation. A higher Child Behaviour Questionnaire score indicates more active and more intense behaviour. A higher Parenting Daily Hassles Tasks scale score indicates more hassle. A higher Hospital Anxiety and Depression Scale score indicates greater symptoms of anxiety/depression. A higher score of Child Health Visual Analogue Scale indicates better health. A higher PedsQL score indicates better quality of life.

¶Missing values refer to those with ≥50% items on any scale missing.

**Missing values refer to those with more than one item missing.

PedsQL, the Pediatric Quality of Life Inventory.

Table 3 shows frequency of exposures and ORs for the CC and MI analyses. Compared with controls, case parents were significantly more likely to leave stair gates open (AOR 3.09, 95% CI 2.39 to 4.00) or to not have stair gates (AOR 2.50, 95% CI 1.90 to 3.29), to not have carpeted stairs (AOR 1.51, 95% CI 1.09 to 2.10) or to not have a landing part-way up their stairs (AOR 1.34, 95% CI 1.08 to 1.65). They were more likely to consider their stairs not safe to use (AOR 1.46, 95% CI 1.07 to 1.99) or in need of repair (AOR 1.71, 95% CI 1.16 to 2.50). Case households were significantly less likely to have tripping hazards on their stairs (AOR 0.77, 95% CI 0.62 to 0.97) or not have handrails on all stairs (AOR 0.83, 95% CI 0.75 to 0.93). The PAF percentage ranged from 5% for various stair features to 18% for not having a landing part-way up their stairs, to 21% for not having a stair gate and to 24% for leaving a safety gate open on stairs.

**Table 3:** Frequency of exposures in case and control participants, adjusted ORs from complete case and multiple imputation analyses and population attributable fraction (PAF) percentage

Exposures	Cases n=610	Controls n=2658	Adjusted OR (95% CI)	PAF (%)	Confounders adjusted for§*
Safety gates anywhere in house†	[12]	[124]	n=1921	–	HADS, PDH, first child, stair safety, hours out-of-home child care
Used	465 (76.3)	2013 (79.4)	1 [reference] 1.22 (0.92 to 1.62)		
Did not use	142 (23.8)	521 (20.6)			

**Exposures only for households with stairs**	**Cases**	**Controls**			
	**n=598**	**n=2476**			
	**[6]**	**[7]**			

Stair gate†	[13]	[40]	n=2401	24 21	Child's ability to open safety gate, taught child rules about going down the stairs, carrying things down the stairs, leaving things on stairs, stair safety
Gate closed	174 (29.7)	1245 (51.1)	1 [reference]		
Gate left open	210 (35.9)	555 (22.8)	3.09 (2.39 to 4.00)		
No gate	201 (34.4)	636 (26.1)	2.50 (1.90 to 3.29)		
Carpeted stairs†	[8]	[28]	n=2394	5	HADS, PDH, stair safety
Had	507 (85.9)	2248 (91.8)	1 [reference]		
Did not have	83 (14.1)	200 (8.2)	1.52 (1.09 to 2.10)		
Landing part-way up the stairs†	[5]	[28]	n=2766	18	Stair safety
Had	180 (30.4)	892 (36.4)	1 [reference]		
Did not have	413 (69.6)	1556 (63.6)	1.34 (1.08 to 1.65)		
Spiral or winding stairs†	[7]	[30]	n=2757	–	Stair safety
Did not have	495 (83.8)	2044 (83.6)	1 [reference]		
Had	96 (16.2)	402 (16.4)	0.97 (0.75 to 1.27)		
Tripping hazards on stairs‡	[18]	[51]	n=2367	–	HADS, PDH, stair safety
Did not have	397 (68.4)	1493 (61.6)	1 [reference]		
Had	183 (31.5)	932 (38.4)	0.77 (0.62 to 0.97)		
Stairs too steep^$^†	[18]	[80]	n=2744	–	Stair safety
Other responses	362 (62.4)	1653 (69.0)	1 [reference]		
Agree	218 (37.6)	743 (31.0)	1.21 (0.94 to 1.56)		
Stairs too narrow^$^†	[23]	[98]	n=2742	–	Stair safety
Other responses	421 (73.2)	1894 (79.7)	1 [reference]		
Agree	154 (26.8)	484 (20.4)	1.28 (0.96 to 1.70)		
Stairs poorly lit^$^†	[26]	[94]	n=2380	–	HADS, PDH, stair safety
Other responses	469 (82.0)	2053 (86.2)	1 [reference]		
Agree	103 (18.0)	329 (13.8)	1.32 (0.97 to 1.79)		
Steps in need of repair^$^†	[25]	[96]	n=2378	5	HADS, PDH, stair safety
Other responses	506 (88.3)	2233 (93.8)	1 [reference]		
Agree	67 (11.7)	147 (6.2)	1.71(1.16 to 2.50)		
Banister/handrail on stairs in need of repair^$^†	[32]	[98]	n=2377	–	HADS, PDH, stair safety
Other responses	498 (88.0)	2175 (91.5)	1 [reference]		
Agree	68 (12.0)	203 (8.5)	1.32 (0.92 to 1.88)		
Stair covering in need of repair^$^†	[26]	[96]	n=2378		HADS, PDH, stair safety
Other responses	501 (87.6)	2205 (92.6)	1 [reference]		
Agree	71 (12.4)	175 (7.4)	1.41 (0.99 to 2.03)		
Stairs safe to use^$^†	[10]	[25]	n=2391	5	HADS, PDH, stair safety
Other responses	487 (82.8)	2180 (88.9)	1 [reference]		
Disagree	101 (17.2)	271 (11.1)	1.46 (1.07 to 1.99)		
Handrails on all stairs^$^†	[1]	[20]	n=2416	–	HADS, PDH, stair safety
Had	382 (64.0)	1393 (56.7)	1 [reference]		
Did not have	215 (36.0)	1063 (43.3)	0.69 (0.56 to 0.86)		
Banisters or railings on all stairs^$^†	[22]	[60]	n=2301	–	HADS, PDH, stair safety
Had	424 (73.6)	1930 (79.9)	1 [reference]		
Did not have	152 (26.4)	486 (20.1)	1.27 (0.99 to 1.63)		
Rules about going down the stairs	[20]	[70]	n=1840	–	HADS, PDH, first child, child's ability to open safety gate, stair gate, stair safety
Had taught child	405 (70.1)	1782 (74.1)	1 [reference]		
Had not taught child	173 (29.9)	624 (25.9)	1.36 (0.92 to 2.02)		
Rules about carrying things while going down the stairs	[20]	[68]	n=1840	–	HADS, PDH, first child, child's ability to open safety gate, stair gate, stair safety
Had taught child	287 (49.7)	1274 (52.9)	1 [reference]		
Had not taught child	291 (50.4)	1134 (47.1)	1.21 (0.83 to 1.75)		
Rules about leaving things on stairs	[22]	[64]	n=1838	–	HADS, PDH, first child, child's ability to open safety gate, stair gate, stair safety
Had taught child	256 (44.4)	1073 (44.5)	1 [reference]		
Had not taught child	320 (55.6)	1339 (55.5)	0.85 (0.60 to 1.22)		

**Exposure only for households with stairs and banisters**	**Cases** **(n=424)**	**Controls (n=1930)**			

Banister width (inches)	[190]	[803]	n=627	–	Stair safety
0–2.5	94 (40.2)	400 (35.5)	1 [reference]		
2.5–3.75	67 (28.6)	363 (32.2)	0.83 (0.53 to 1.29)		
≥3.75	73 (31.2)	364 (32.3)	0.75 (0.48 to 1.18)		

**Exposures only for children aged 0–36 months**	**Cases** **(n= 475)**	**Controls** **(n=2009)**			

Baby walker	[14]	[32]	n=1620	–	HADS, PDH, first child, hours out-of-home child care
Did not use	326 (70.7)	1302 (65.9)	1 [reference]		
Used	135 (29.3)	675 (34.1)	0.83 (0.63 to 1.10)		
Playpen or travel cot	[14]	[30]	n=1615	–	HADS, PDH, used baby walker, first child, hours out-of-home child care
Used	77 (16.7)	334 (16.9)	1 [reference]		
Did not use	384 (83.3)	1645 (83.1)	1.07 (0.75 to 1.53)		
Stationary activity centre	[16]	[33]	n=1611	–	HADS, PDH, used baby walker, first child, hours out-of-home child care
Used	111 (24.2)	490 (24.8)	1 [reference]		
Did not use	348 (75.8)	1486 (75.2)	1.08 (0.80 to 1.46)		

[ ] missing values.

Stair safety is a composite variable combining responses to questions marked with $ and grouped as all ‘safe’ responses, some ‘safe’ responses and no ‘safe’ responses. Where the exposure variable is a measure of stair safety, this variable is excluded from the composite stair safety measure used as a confounder in adjusted analyses.

*Conditional logistic regression excludes observations where all cases and their matched controls have the same exposure.

†In the past 24 h.

‡In the past week.

§All models were adjusted for the Index of Multiple Deprivation and distance from the hospital plus listed confounders.

HADS, Hospital Anxiety and Depression Scale; PAF, population attributable fraction; PDH, Parenting Daily Hassles Scale.

AORs from the MI analyses differed only by >10% from the CC analyses for four exposures (not having carpeted stairs (11% higher in MI than CC analysis), stair covering in need of repair (11% higher in MI than CC analysis), banister width ≥3.75 inches (20% higher in MI than CC analysis) and use of stationary activity centre (11% lower in MI than CC analysis)).

There were several significant interactions (see online supplementary table S1). Compared with having a stair gate that was kept closed, leaving stair gates open increased the odds of a stair fall injury in those aged 0–36 months, with a particularly high odds of injury among children aged 0–12 months. Not having a stair gate increased the odds of a stair fall injury in all age groups, again with higher odds in younger children, but the difference between age groups was less marked than for leaving gates open. The relationship between stair gate use and stair fall injuries also differed between families who used and did not use baby walkers. Leaving stair gates open or not having a stair gate increased the odds of injury (compared with having a closed stair gate) among walker users and non-users, but the odds of injury were particularly high in walker users who left gates open. There were significant interactions between the number of adults in paid work and teaching rules about (1) carrying things while going down the stairs and (2) leaving things on stairs. Not teaching children either rule reduced the odds of a stair fall injury in families where none of the adults were employed but not in families with employed adults. There were also significant interactions between the number of adults in the household and two exposures. There was a reduced odds of a stair fall injury in single adult households not teaching rules about leaving things on stairs but not in households with two or more adults. There was an increased odds of a stair fall injury in single adult households without carpeted stairs but not in households with two or more adults.

## Discussion

A range of factors, most of which were modifiable, increased the odds of stair falls in children aged 0–4 years. This included not using safety gates on stairs or leaving gates open, particularly in families also using baby walkers, not having carpet on stairs, not having a landing part-way up the stairs, having stairs that were in need of repair or having stairs that parents perceived to be unsafe. The PAF ranged from 5% to 24% for these factors individually, but 45% of stair fall injuries could be prevented by having stair gates and keeping them closed assuming our associations are causal.

### Strengths and limitations

This large case–control study took place in English NHS hospitals and included both urban and rural areas with varied levels of socioeconomic deprivation (ranging from 10% of population living in the 20% most deprived areas in England for Norfolk to 52% for Nottingham).^22^ We adjusted for a wide range of potential confounding factors selected using directed acyclic graphs. Analyses using multiply imputed data revealed broadly similar findings to CC analyses.

Our participation rate was low (33% of cases and 29% of controls). Participation rates were similar by sex of child, but a higher proportion of participants were aged 0–12 months than non-participants. We could not collect exposure data from non-participants and hence the extent to which selection bias may have occurred is unknown. Self-reported exposures may have been subject to recall or social desirability bias. Our cases were more disadvantaged than controls, and some exposures may have been associated with disadvantage. Although we adjusted for area level deprivation, some residual confounding may have remained. Failure to find significant associations for exposures whose prevalence was outside the range used in our sample size calculation (table 3; spiral/winding stairs, narrow stairs, poorly lit stairs, banister/handrail in need of repair, use of playpen or stationary activity centre) may be due to insufficient power.

We found several ‘counter-intuitive’ findings. Children living in houses without handrails on all stairs and those with tripping hazards on stairs had lower odds of injury. It is possible that if parents perceive stairs to be unsafe they may restrict access to the stairs or supervise children differently. Our findings regarding the increased odds of a fall associated with teaching children safety rules in families with unemployed or single parents may reflect the poorer quality and more hazardous housing in which such families may live,^23^ and despite adjusting for a range of confounders, this may not have been taken fully into account in our analyses. As we explored associations between many exposures and falls, some significant findings may represent type 1 errors.

### Comparisons with existing literature

We have found only one small Australian case–control study of infants with head or face injuries with which to compare our findings. The study examined associations between use of safety gates and falls in families using baby walkers, and found that households using baby walkers without stair guards or barriers had a 3.5-fold increased risk of child head injury than those using guards or barriers (OR 3.53, 95% CI 1.21 to 10.30).^24^ Our study extends the findings of the Australian study by showing that the odds of a stair fall were particularly high in families who used baby walkers and left stair gates open. This may be partly explained by risk compensation if families who use safety gates use walkers upstairs more often, feel ‘safer’ using walkers upstairs or forget to close gates more often.

## Conclusion

If the associations we found are causal, use of safety gates on stairs, not leaving safety gates open, particularly in families also using baby walkers, using carpet on stairs, keeping stairs in good repair and having a landing part-way up the stairs could individually prevent between 5% and 24% of injuries from falls on stairs and, if families had stair gates and kept them closed, 45% of injuries could be prevented. This advice could be included in child health promotion programmes, personal child health records, home safety assessments and other child health contacts. Future research is needed to explore associations between some stair characteristics, use of playpens and stationary activity centres and injury occurrence.

## Supplementary Material

Web table
