# Supplementary material for: Risk and protective factors for falls on stairs in young children: multicentre case–control study
Source: Arch Dis Child. 2015 Dec 10;101(10):909–16. doi: 10.1136/archdischild-2015-308486 (PMC5050281; doi:10.1136/archdischild-2015-308486)
Supplement: Web table [file archdischild-2015-308486-s1.pdf]

Online table1. Significant interactions in adjusted analyses comparing cases with controls

**Exposure**	**Adjusted odds ratios (95% CI) by age group**			**Test for interaction**
	**0-12 months**	**13-36 months**	**≥37 months**	
Stair gate left open[a]	8.64 (3.99, 18.68)	2.64 (1.92, 3.64)	1.52 (0.76, 3.03)	0.008
No stair gate[a]	3.27 (1.48, 7.20)	2.33 (1.60, 3.39)	2.08 (1.23, 3.51)	

	**Adjusted odds ratios (95% CI) by use of baby walker**		**Test for interaction**
	**Used walker**	**Did not use walker**	
Stair gate left open[a]	7.37 (4.36, 12.45)	2.65 (1.87,3.76)	0.002
No stair gate[a]	2.54 (1.33, 4.87))	2.42 (1.63, 3.59)	

	**Adjusted odds ratios (95% CI) by adults in paid work**			**Test for interaction**
	**2 or more**	**One**	**None**	
Not taught child rules about carrying things down stairs	1.45 (0.94, 2.24)	1.26 (0.76, 2.09)	0.44 (0.20, 0.96)	0.009
Not taught child rules about leaving things on stairs	1.01 (0.66, 1.56)	0.88 (0.54, 1.42)	0.27 (0.12, 0.60)	0.004

	**Adjusted odds ratios (95% CI) by number of adults living with child**		**Test for interaction**
	**One adult**	**More than one adult**	
Not taught children rules about leaving things on stairs	0.33 (0.15, 0.75)	0.95 (0.66, 1.38)	0.01
Stairs not carpeted[a]	11.07 (3.89, 31.53)	1.15 (0.79, 1.66)	<0.001

Adjusted for confounders in table 3. [a] in the last 24 hours.
